# A commentary on ‘Exosomes miRNA-499a-5p targeted CD38 to alleviate anthraquinone induced cardiotoxicity: experimental research’

**DOI:** 10.1097/JS9.0000000000001629

**Published:** 2024-05-17

**Authors:** Yueh-Wei Liu, Ching-Hua Hsieh

**Affiliations:** a Department of General Surgery, Kaohsiung Chang Gung Memorial Hospital, and Chang Gung University, College of Medicine; b Department of Trauma Surgery, Kaohsiung Chang Gung Memorial Hospital, and Chang Gung University, College of Medicine, Kaohsiung, Taiwan

*Dear Editor,*

We have read the recent article ‘Exosomes miRNA-499a-5p targeted CD38 to alleviate anthraquinone induced cardiotoxicity: experimental research’^1^ in the *International Journal of Surgery* with great interest. In appreciation for the extensive research into the role of exosomal miRNA-499a-5p in curing doxorubicin (DOX)-induced cardiotoxicity, I would like to express a few concerns about several of the descriptions in this article.

First, some descriptions and errors were found in the text about Figure 2. The description in the text regarding Figure 2D–F is about the double luciferase activity, which is shown in Figure 2E. Regarding Figure 2F, the results showed that miRNA-499-5p can decrease CD38 protein expression in CD38-mut but not CD38-wt mice, which contradicts the evidence in Figure 2D. Second, the label in Figure 5B, C regarding C-B-exo-miRNA-499a-5p was not revealed correctly. In addition, there is no Figure 5D, as stated in the legend. Third, the indexing of the treatment in Figure 7A was incorrect. In the third and fourth groups, the DOX label should be designated as ‘+.’ This will ensure that the treatment for DOX-induced cardiotoxicity aligns appropriately with the descriptions provided in the bar figure. Similar errors were identified in Figures 7G, 9A, E, I, 11A, E, and 12H. Fourth, the author demonstrated the characterization of B-exo, but not the designed C-B-exo. The transfer of miRNAs via electroporation would have had a significant impact on exosome integration and should be demonstrated. Furthermore, the Western blot of the exosomal surface marker did not disclose a negative control, such as calnexin or GM130, which eliminated the possibility of contamination^2^. Furthermore, bioinformatics analysis using miRbase and TargetScan revealed that a potential target of miRNA-499a-5p is not only CD38 but many other candidates. Actually, there were more targets found by the combined approaches with these two algorithms; the author did not state clearly the reason or consideration for choosing CD38 as the target. Notably, in the legend of Figure 2, the author describes that the bioinformatics analysis is based on four predictive algorithms, including miRbase, TargetScan, microRNA.org, and starBase v2.0. Finally, anthraquinone and DOX are related compounds with distinct characteristics. In terms of medical significance, DOX is commonly used in cancer treatment, while anthraquinone derivatives like mitoxantrone are associated with more cardiotoxic risk than DOX. Seeing all the studies performed in this article are based on DOX, I think the title using anthraquinone may not be suitable for the use of DOX. Our suggestions are just to enhance an already excellent piece of study. We eagerly await additional detailed information from the authors. Thank you.

## Ethical approval

Not applicable.

## Consent

Not applicable.

## Sources of funding

The funding for this research was provided by the Chang Gung Memorial Hospital through grant number CMRPG8N0111, awarded to Yueh-Wei Liu.

## Author contribution

Y.-W.L.: manuscript writing; C.-H.H.: study concept and literature reviewing.

## Conflicts of interest disclosure

The authors declare no conflicts of interest.

## Research registration unique identifying number (UIN)

None.

## Guarantor

Both authors.

## Data availability statement

Not applicable.

## Provenance and peer review

Not commissioned, externally peer-reviewed.

## References

[1] Ma C Yang Z Wang J . Exosomes miRNA-499a-5p targeted CD38 to alleviate anthraquinone induced cardiotoxicity: experimental research. Int J Surg 2024;110:1992–2006. 38277348 10.1097/JS9.0000000000001118 PMC11019978

[2] Welsh JA Goberdhan DCI O’Driscoll L . Minimal information for studies of extracellular vesicles (MISEV2023): from basic to advanced approaches. J Extracell Vesicles 2024;13:e12404. 38326288 10.1002/jev2.12404 PMC10850029

